# Characterization of the complete mitochondrial genome of *Abscondita cerata* (Olivier, 1911) (Coleoptera: Lampyridae) and its phylogenetic implications

**DOI:** 10.1080/23802359.2021.1959456

**Published:** 2021-07-28

**Authors:** Liang-Jong Wang, Yu-Wei Wu, Tzi-Yuan Wang

**Affiliations:** a Division of Forest Protection, Taiwan Forestry Research Institute, Taipei, Taiwan; b Graduate Institute of Biomedical Informatics, College of Medical Science and Technology, Taipei Medical University, Taipei 106, Taiwan; c Biodiversity Research Center, Academia Sinica, Taipei, Taiwan

**Keywords:** Mitochondrial genome, *Abscondita cerata*, Lampyridae, Coleoptera, next-generation sequencing

## Abstract

We sequenced and assembled the complete mitochondrial genome of *Abscondita cerata* from Nankang, Taipei City, Taiwan. The complete mitogenome of *A. cerata* is 16,964 bp long, and contains 13 protein-coding, 22 tRNA, and two rDNA genes. Nucleotide compositions of the mitogenome of the *A. cerata* are A: 43.93%, T: 36.74%, C: 11.05%, and G: 8.28%. The AT and GC skewness of the mitogenome sequence are 0.0891 and −0.1434, showing the genome composition skewness toward adenine and cytosine. The clade including all Lampyridae species is well supported. The result indicates that Luciolinae is a monophyletic group but Lampyrinae is not a monophyletic group, as *Lamprigera yunnana*, which was originally classified into Lampyrinae, is sister to Luciolinae. The genus *Lamprigera* may share a unique phylogenetic position in Lampyridae. The genus *Luciola* is a polyphyletic group and the genus *Abscondita* is a monophyletic group. *A. cerata* is the sister species to *A. chinensis* in China. Mitogenomic data from this study will provide useful molecular markers for further studies on the population genetics, speciation, and conservation of endemic species *A. cerata* in Taiwan.

Lampyridae Rafinesque, 1815, popularly known as fireflies, contains a cosmopolitan group of beetles with approximately 2000 species assigning to 83 genera (Branhm 2010). The genus *Luciola* Laporte is a diverse group containing 283 species (McDermott 1966), and many studies based on morphological characters and DNA data revealed that it is not a monophyletic group (Jusoh et al. 1975; Ballantyne and Lambkin 2001; Chen et al. 2019). After the phylogenetic analysis of the species in SE Asian *Luciola* (sensu lato) by morphological characters, the genus *Abscondita* was erected and six *Luciola* species were transferred to it (Ballantyne et al. 2013). Subsequently, two new species of *Abscondita* were described and *Luciola pallescens* Gorham was transferred to *Abscondita*, making a total of nine species assigned to the genus (Ballantyne et al. 2019). *Abscondita cerata* (Olivier, 1911) is endemic to Taiwan and it is the only species of *Abscondita* restricted to one island (Ballantyne et al. 2013). *Abscondita cerata* ranges widely from sea level to 1500 m a.s.l. in Taiwan. Its flight period is mainly from March to May (Chen 2003). This is the first report of complete mitochondrial sequences for the species.

Seventeen specimens of *A. cerata* in this study were collected in Nankang, Taipei City, Taiwan (25°01′40.4″N 121°38′02.6″E) in April 2019. Total genomic DNA of the male specimen was extracted from the thorax of the adult using a ZR Tissue & Insect DNA MicroPrep™ kit (D6015) following the supplier’s instructions. A specimen was deposited at Biodiversity Research Center, Academia Sinica, Taipei, Taiwan (https://www.biodiv.tw/, contact person: T. Y. Wang, tziyuan@gmail.com) under the voucher number 190407NKfly_BM8. The voucher specimen and the other specimens collected in the same site were identified to species level by L. J. Wang based on the references (Chen 2003; Ballantyne et al. 2013). The complete mitogenome of *A. cerata* was sequenced using the next-generation sequencing method: the sheared DNA fragments were used for library construction with the MGIEasy DNA Library Prep Kit v1.1. The library was sequenced with the BGISEQ-500RS sequencer by Tri-I Biotech (New Taipei, Taiwan). A total of 9 Gb next-generation sequencing paired-end reads were used to assemble the complete mitogenome sequence. The CLC Genomics Workbench ver.12.0.02 (QIAGEN, Hilden, Germany) was used for sequence quality analysis, data trimming, and *de novo* assembly by default setting. The assembled contigs were then used to BLAST against the mitogenomes of the congeners *A. anceyi* (NCBI acc. MH020192) and *A. terminalis* (NCBI acc. MK292092) to identify the contig with the highest BLAST score and lowest e-value as the mitogenome of *A. cerata*. The locations of the protein-coding genes, ribosomal RNAs (rRNAs), and transfer RNAs (tRNAs) were predicted using MITOS Web Server (Bernt et al. 2013) followed by alignment with other mitogenomes of fireflies in the family Lampyridae. The AT and GC skew was calculated according to the following formulas: AT skew=(A – T)/(A + T) and GC skew=(G – C)/(G + C) (Perna and Kocher 1995). Maximum-likelihood (ML) analyses were performed using the GTRGAMMA model implemented in RAxML v.8.1.17 (Stamatakis 2014). Nodal support confidence was estimated using a fast bootstrapping analysis with 1000 replicates in RAxML with the model GTRCAT.

The complete mitogenome of *A. cerata* is 16,964 bp in length (GenBank accession no. MW751423), including 13 protein-coding genes, two rRNA genes, 22 tRNA genes, and one control region. The total nucleotide compositions of the *A. cerata* mitogenome are 43.93% for A, 36.74% for T, 11.05% for C, and 8.28% for G. The AT and GC skewness of the mitogenome sequence are 0.0891 and −0.1434, showing the genome composition skewness toward adenine and cytosine. The gene rearrangement of the mitogenome in *A. cerata* is identical to the ancestral inferred insect type (Cameron 2014). We reconstructed the phylogenetic relationships including 28 Lampyridae species and two outgroups *Rhagophthalmus ohbai* (Rhagophthalmidae) and *Chauliognathus opacus* (Cantharidae) based on 13 mitochondrial protein-coding genes (Figure 1). Bootstrap values are shown at the branch nodes. The clade consisting of all Lampyridae species is well supported (100%). The phylogenetic tree shows that *Lamprigera yunnana*, which was originally classified in Lampyrinae (McDermott 1966), is sister to Luciolinae. This result is consistent with the molecular phylogenetic result of Chen et al. (2019), indicating that Luciolinae is a monophyletic group while Lampyrinae is not monophyletic. The genus *Lamprigera* may share a unique phylogenetic position in Lampyridae. The genus *Luciola* is a polyphyletic group although *Luciola substriata* have been transferred to the newly erected genus *Sclerotia* (Ballantyne 2016). The clade containing *A. cerata* (MW751423), *A. anceyi* (MH020192), *A. chinensis* (MK122952), and *A. terminalis* (MK292092) receives absolute support (100%), confirming the genus *Abscondita* is a monophyletic group. *A. cerata* is the sister species to *A. chinensis* in China. More complete mitogenomic data from other Lampyridae species are needed for further studies on the phylogeny of Lampyridae. Mitogenomic data from this study will provide useful molecular markers for further studies on the population genetics, speciation, and conservation of endemic species *A. cerata* in Taiwan.

**Figure 1.**
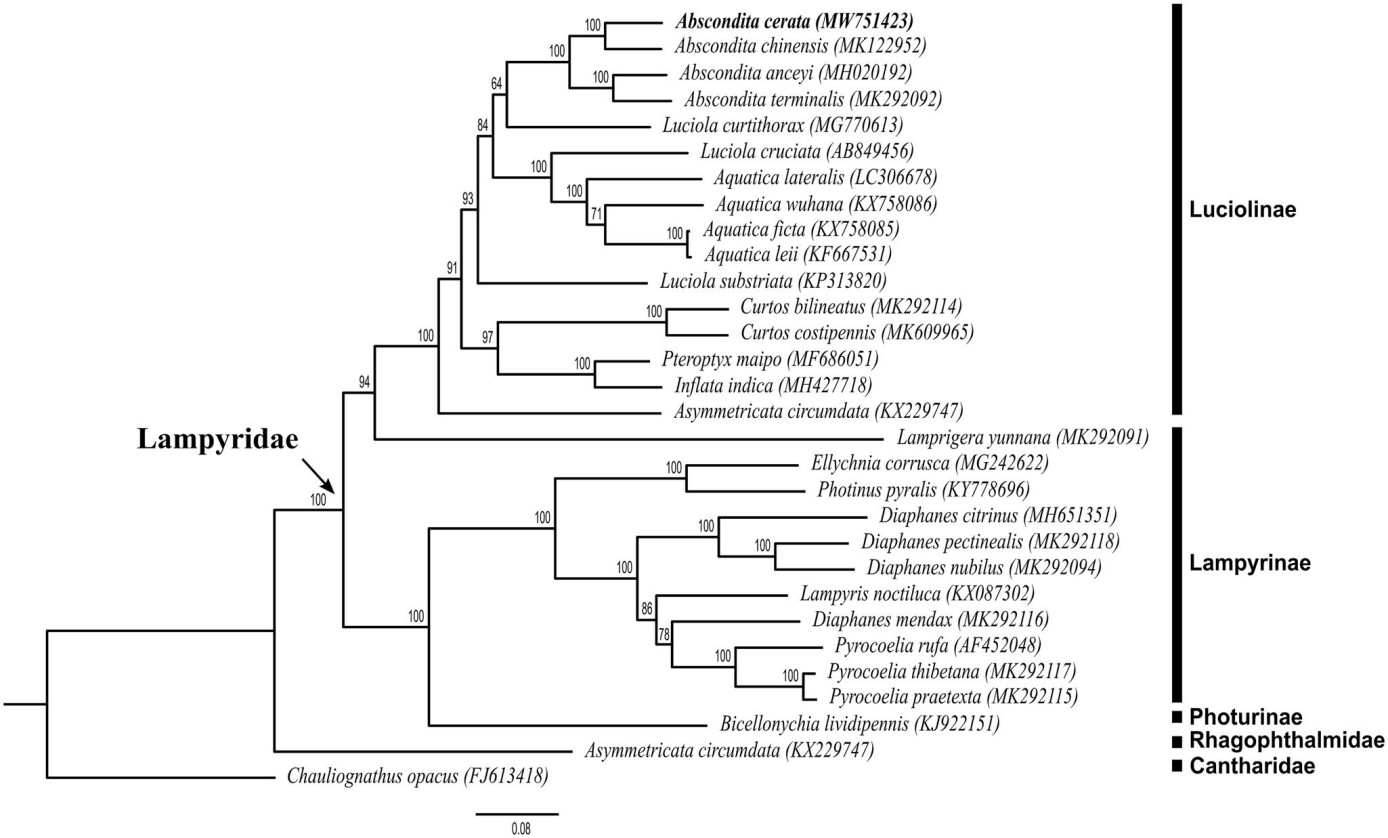
Phylogenetic tree of 28 Lampyridae species including *Abscondita cerata* (in this study, MW751423) and 2 outgroups based on the sequence of mitochondrial 13 protein-coding genes. The tree was reconstructed under the GTRGAMMA model implemented in RAxML v.8.1.17 (Stamatakis 2014). Nodal support confidence was estimated using a fast bootstrapping analysis with 1000 replicates in RAxML with the model GTRCAT.

## Data Availability

The genome sequence data that support the findings of this study are openly available in GenBank of NCBI (National Center for Biotechnology Information) at https://www.ncbi.nlm.nih.gov under the accession no. MW751423. The associated BioProject, SRA, and Bio-Sample numbers are PRJNA699421, SRR13626958, and SAMN17776035, respectively.

## References

[CIT0001] Ballantyne L, Fu XH, Lambkin C, Jeng ML, Faust L, Wijekoon WMCD, Li DQ, Zhu TF. 2013. Studies on South East Asian fireflies: *Abscondita*, a new genus with details of life history, flashing patterns and behaviour of *Abs. chinensis* (L.) and *Abs. terminalis* (Olivier) (Coleoptera: Lampyridae). Zootaxa. 3721(1):1–48. 26120657 10.11646/zootaxa.3721.1.1

[CIT0002] Ballantyne L, Lambkin C. 2001. A new firefly, *Luciola* (Pygoluciola) *kinabalua* sp. nov. (Coleoptera: Lampyridae), from Malaysia, with observations on a possible copulation clamp. Raffles Bull Zool. 49(2):363–377.

[CIT0003] Ballantyne LA, Lambkin CL, Ho JZ, Jusoh WFA, Nada B, Nak-Eiam S, Thancharoen A, Wattanachaiyingcharoen W, Yiu V. 2019. The Luciolinae of S. E. Asia and the Australopacific region: a revisionary checklist (Coleoptera: Lampyridae) including description of three new genera and 13 new species. Zootaxa. 4687(1):zootaxa.4687.1.1. 31719466 10.11646/zootaxa.4687.1.1

[CIT0004] Ballantyne LA, Lambkin CL, Luan X, Boontop Y, Nak-Eiam S, Pimpasalee S, Silalom S, Thancharoen A. 2016. Further studies on South Eastern Asian Luciolinae: 1. *Sclerotia* Ballantyne, a new genus of fireflies with back swimming larvae 2. *Triangulara* Pimpasalee, a new genus from Thailand (Coleoptera: Lampyridae). Zootaxa. 4170(2):201–249. 27701260 10.11646/zootaxa.4170.2.1

[CIT0005] Bernt M, Donath A, Jühling F, Externbrink F, Florentz C, Fritzsch G, Pütz J, Middendorf M, Stadler PF. 2013. MITOS: improved de novo metazoan mitochondrial genome annotation. Mol Phylogenet Evol. 69(2):313–319. 22982435 10.1016/j.ympev.2012.08.023

[CIT0006] Branhm MA. 2010. Lampyridae Latreille, 1817, pp. 141–149. In: Leschen RAB, Beutel RG, Lawrence JF, editors. Coleoptera, beetles. Volume 2: morphology and systematics (Elateroidea, Bostrichiformia, Cucujiformia partim). Berlin, Germany: Walter de Gruyter.

[CIT0007] Cameron SL. 2014. Insect mitochondrial genomics: implications for evolution and phylogeny. Annu Rev Entomol. 59:95–117. 24160435 10.1146/annurev-ento-011613-162007

[CIT0008] Chen TR. 2003. The fireflies of Taiwan. Taipei: Field Image Publisher.

[CIT0009] Chen X, Dong Z, Liu G, He J, Zhao R, Wang W, Peng Y, Li X. 2019. Phylogenetic analysis provides insights into the evolution of Asian fireflies and adult bioluminescence. Mol Phylogenet Evol. 140:106600. 31445200 10.1016/j.ympev.2019.106600

[CIT0011] Jusoh WFA, Ballantyne L, Chan SH, Wong TW, Yeo D, Nada B, Chan KO. 1975. Molecular systematics of the firefly genus Luciola (Coleoptera: Lampyridae: Luciolinae) with the description of a new Species from Singapore. Biochem Pharmacol. 24(17):1639–1641. 33806564 10.3390/ani11030687 PMC7998795

[CIT0012] McDermott FA. 1966. Lampyridae. In: Steel WO, editor. Coleopterorum Catalogus Supplementa. Pars 9. Editio Secunda. S’Gravenhage, The Netherlands: W. Junk; p. 149.

[CIT0013] Perna NT, Kocher TD. 1995. Patterns of nucleotide composition at fourfold degenerate sites of animal mitochondrial genomes. J Mol Evol. 41(3):353–358. 7563121 10.1007/BF00186547

[CIT0014] Stamatakis A. 2014. RAxML version 8: a tool for phylogenetic analysis and post-analysis of large phylogenies. Bioinformatics. 30(9):1312–1313. 24451623 10.1093/bioinformatics/btu033 PMC3998144

